# A New Form of Treatment in Uterine Disease

**Published:** 1898-02

**Authors:** Herman D. Marcus

**Affiliations:** Philadelphia, Pa.; Late Lecturer on Materia Medica and Therapeutics at the Medico-Chirurgical College


### A NEW FORM OF TREATMENT IN UTERINE DISEASE.

By HERMAN D. MARCUS, M.D., PHILADELPHIA, PA.

Late Lecturer on Materia Medica and Therapeutics at the Medico-Chirurgical College.

Discoveries of new remedial agents have during the past decade become so excessively abundant that it is impossible to give each and every one a thorough clinical trial. The majority of these new drugs or new combinations are quickly discarded owing to their failure to overcome diseased conditions any better than old reliable formulas. When, then, a preparation is placed on the market that fulfils freely indications for which they may be applied, it behooves practitioners to at least give such a combination a thorough trial without regard to any objection on the plea of code or ethics. Our patient's welfare should above everything be our first consideration, and should not suffer any neglect by reason of the mistaken duty of the physician towards the profession.

There are few conditions which tax the practitioner to greater effort than diseases of the female generative organs. A woman suffering from uterine, ovarian or vaginal disease is not only a burden on herself, but is a burden also to her family and friends. Always peevish, fretful and morose, exaggerating every symptom until, perhaps, the mental equilibrium becomes also lost, For these conditions a preparation has been placed some years ago upon the market, and owing to the fact that its composition is secret, I, with probably many others, refused to accept it or give it a clinical trial. I refer to the Micajah uterine wafers.

About three years ago a lady about thirty-five years of age presented herself at my office for treatment giving following history of her case: She had been fourteen years married; had never any living children, but two miscarriages, one twelve years before at four months, and the other ten years ago at five months; a year before calling on me she began to notice a profuse leucorrhea of a thick yellow character, and each menstruation profuse, thick and black in character; menstruated irregularly, at times every three weeks and then again, perhaps, every two weeks; had dragging sensation in pelvis "just as if the womb would fall out"; pain in lumbar region; a continual desire to urinate, at times urinating fifteen to twenty times daily. On digital examination of the parts the uterus was found enlarged, thickened and considerably anteverted, in fact so much that the fundus was pressing against the bladder. From the os a thick pus-like discharge exuded. In the vagina a large ulcer was observed. Diagnosis of chronic metritis and endometritis was made and the usual treatment prescribed. Hot antiseptic (creoline) douches twice daily. Ichthyol tampons and divers other usual remedies were prescribed. The result after two months treatment a distinct failure.

Just about this time my attention was directed toward the Micajah uterine wafers. A sample as usual sent out by drug houses was left with me, and, instead of throwing it as usual in the waste-basket, I concluded to give it a trial, not because I believed it of any value, simply because I had tried everything else. I directed the patient to introduce into her vagina one wafer nightly and to return in one week. The condition of the patient after the firs week was most remarkable. The uterus was decidedly smaller, the discharge less thick and free, the dragging sensation had improved, the urine could be retained much longer, and the desire to urinate frequently was not quite so severe. I ordered her then to use a wafer only every second night for a week and on her return all symptoms were improved. Improvement continued until when discharged forty-two days after using the first wafer the menstruation seemed more normal in character, the uterus became nearly normal in size and only slightly anteverted; absolutely no bladder trouble; the pain in lumbar region and dragging sensation had completely disappeared.

Of course, such a distinctly favorable result quite naturally elated me and I decided to give these wafers a thorough trial. My experience with these wafers dates now since that time (three years) and extends over a variety of uterine and vaginal diseases. *In toto* I have treated with these wafers since then seventeen cases of cervical endometritis, five cases of general endometritis, four cases of metritis, ten cases of dysmenorrhea complicated with endometritis, post abortive. Of the total of fifty-five cases, thirty-eight were cured, nine cases improved, and the balance remained unimproved, a percentage of cures larger than for any other form of treatment. Some twenty-five or thirty cases of leucorrhea treated with the wafers showed cures in from three to six weeks.

There is neither a desire on my part to laud any certain remedy, nor am I optimistic with reference to any form of treatment, but results on cases not selected but taken at random and compared with others prompt me to say that in my opinion these wafers are unquestionably curative in the disease indicated, act more promptly and can be applied with greater ease by the patient than any other form of treatment recommended.

